# Neuromuscular control strategies underpinning motor expertise in yoga: a biomechanical analysis of twisting poses in expert and novice practitioners

**DOI:** 10.3389/fpsyg.2026.1715999

**Published:** 2026-03-04

**Authors:** Lijun Hua, Chunlin Luo, Gengchao Bi

**Affiliations:** 1 College of Physical Education and Training, Harbin Sport University, Harbin, China; 2 Graduate School, Harbin Sport University, Harbin, China

**Keywords:** motor expertise, neuromuscular control, surface electromyography (sEMG), twist pose, yoga

## Abstract

**Background:**

This study investigates the neuromuscular control strategies and motor expertise underlying complex yoga movements by comparing expert (*n* = 17) and novice (*n* = 17) female practitioners during two twisting poses: the Standing Twist (ST) and the Semi-Triangle Twist (STT).

**Methods:**

Utilizing musculoskeletal modeling and surface electromyography (sEMG), we examined how internal coordination adapts to varying task demands.

**Results:**

While the STT imposed significantly higher mechanical demands—evidenced by greater peak trunk rotation (*p* < 0.001), higher peak moments (*p* = 0.020), and increased erector spinae activation—the external kinematic performance did not distinguish experts from novices. Crucially, expertise was manifested at the neuromuscular level: experts exhibited a refined recruitment pattern characterized by higher rectus abdominis activation compared to novices (unadjusted *p* < 0.05), suggesting a more targeted core stabilization strategy. Furthermore, the lower-load ST pose induced a significantly higher lumbar co-activation ratio (*p* = 0.015), indicating that muscular coordination efficiency is non-linearly related to task difficulty.

**Conclusion:**

These findings suggest that motor expertise in yoga is not defined by observable movement outcomes, but by the optimization of internal motor representations and the strategic recruitment of musculature to manage spinal stability.

## 1 Introduction

Yoga, as a sophisticated mind–body practice, offers a unique window into the study of complex motor control and neuromuscular adaptation (Field, 2011; Telles et al., 2021) and enhance practitioners’ flexibility, strength, and balance (Shin, 2021). Beyond its documented benefits for physical and mental health (Shin, 2021), yoga practice requires high levels of proprioception, balance, and the precise management of degrees of freedom (DoF) within the musculoskeletal system (Jeter et al., 2014). Additionally, it has shown positive effects in the prevention of chronic diseases such as knee osteoarthritis and low back pain (Kuntz et al., 2018), (Wieland et al., 2022). Among the many asanas, twisting poses have received considerable attention for their potential benefits to spinal health. Relevant literature indicates that twisting poses can increase spinal flexibility and strengthen the surrounding muscle groups, which are essential for the functional maintenance of spinal stability (Williams et al., 2005). Modern sedentary lifestyles pose a serious threat to spinal health. Research has shown that prolonged static axial loading can lead to posterolateral damage of the intervertebral disks, increasing the risk of disk herniation (Paul et al., 2017). In this context, yoga twists serve as a functional motor task that potentially optimizes the internal motor representations of the spine. Furthermore, a decline in trunk rotational ability is closely associated with an increased risk of falls (Ljungquist et al., 2003; Mok et al., 2004). Therefore, against the backdrop of increasingly prevalent sedentary behavior, using yoga twist poses as sophisticated neuromuscular training paradigm for enhancing motor coordination and preserving trunk mobility holds significant practical importance (Paul et al., 2017).

Despite yoga’s numerous benefits, improper practice carries a risk of injury, and twisting poses are considered a high-risk category due to the complex neuromuscular coordination required (Lee et al., 2019). The execution of yoga twists involves complex 3D spinal kinematics, where insufficient neuromuscular coordination can lead to suboptimal loading on the intervertebral disks (Longpré et al., 2015; Liu et al., 2021), leaving a gap in the systematic investigation of the neuromuscular control mechanisms and motor strategies unique to twisting asanas. To systematically evaluate the biomechanical characteristics and potential risks of twisting poses, a comprehensive understanding of spinal flexibility and stability is necessary. Flexibility ensures the trunk has a normal range of motion and is often quantified by the trunk rotation angle. Stability, on the other hand, reduces injury risk by optimizing muscle activation patterns (Micheo et al., 2012) and is closely related to the level of muscle activation, particularly of the deep muscles. Meanwhile, the trunk rotation moment can reflect the pressure experienced by the intervertebral disks during movement (Kuai et al., 2017). Among these, deep muscles such as the transversus abdominis and multifidus are primarily responsible for maintaining spinal stability, and their activation patterns are relatively independent of the direction of movement, whereas superficial muscles are more involved in executing trunk rotation movements (Hodges, 2003).

To explore the underlying motor control mechanisms of yoga twists, this study selected two representative poses with distinct task constraints: the Standing Twist (ST) and the Semi-Triangle Twist (STT). The fundamental difference between the two lies in the trunk posture during the twist: ST is performed in an upright, neutral position, whereas STT requires execution in a state of deep hip flexion. We hypothesized that this difference in foundational posture would necessitate a distinct neuromuscular signature, characterized by significant changes in spinal load distribution and the coordination strategies of core muscles. A central question in motor learning is how motor expertise influences the recruitment of musculature to achieve similar kinematic goals (Seth et al., 2018; Navacchia et al., 2019). This study, therefore, investigates whether these neuromuscular control strategies diverge between practitioners of varying yoga expertise when facing these distinct task demands. To comprehensively analyze these differences, this study employed a method combining musculoskeletal (MSK) modeling and simulation with surface electromyography (sEMG). The OpenSim MSK model can estimate internal variables not directly obtainable through standard three-dimensional motion analysis, such as joint moments, thereby providing a basis for assessing joint loads (Seth et al., 2018). sEMG technology, in turn, simultaneously reveals the neuromuscular control strategies employed by muscles to complete the movement (Navacchia et al., 2019). This integrated analytical approach provides a powerful tool for comprehensively understanding the biomechanical characteristics of yoga poses.

Accordingly, the primary objective of this study is to compare the biomechanical and neuromuscular characteristics—specifically trunk rotation angle, moment, and core muscle activation patterns—between ST and STT, and to investigate how these patterns differ across practitioners of varying yoga expertise. We proposed the following hypotheses: (1) Due to the additional forward-flexed posture, STT will generate a larger trunk rotation moment and higher levels of lumbar muscle activation; (2) Compared to novices, experts will demonstrate expertise-driven optimization of muscle activation patterns, specifically characterized by refined motor coordination and lower levels of antagonist muscle co-activation.

## 2 Methods

### 2.1 Participants

An *a priori* sample size calculation was performed using G*Power software (version 3.1.9.7) to ensure sufficient statistical power. Based on a two-way mixed-design analysis of variance (within-between subjects interaction), with a set medium effect size (*f* = 0.25), an alpha level of 0.05, and a desired power of 0.8, the analysis indicated that a total of 34 participants were required. This corresponds to a requirement of 17 participants for each group (expert and novice). We recruited 17 female participants for the expert group, who had over 3 years of practice experience, with an age of 20.50 ± 0.58 years, height of 1.64 ± 0.06 m, and weight of 52.13 ± 4.33 kg. The novice group consisted of 17 female participants with no yoga experience, with an age of 21.50 ± 3.79 years, height of 1.66 ± 0.02 m, and weight of 52.00 ± 3.56 kg. Inclusion criteria were no history of sports injuries within the past year, no history of spinal diseases, and good physical health. This study was approved by the Ethics Committee of Harbin Sport University (Approval No. 2024012), and all participants signed a written informed consent form before the tests.

### 2.2 Testing protocol

Eight high-speed cameras (Qualisys, Sweden; sampling frequency: 200 Hz) were used to capture kinematic data. Synchronized ground reaction force data were collected using two three-dimensional force plates with built-in amplifiers (AMTI-0.5 m, AMTI, United States; dimensions: 60 × 40 × 10 cm; sampling frequency: 100 Hz). Muscle activity data were recorded using a wireless surface electromyography (sEMG) system (Trigno^®^, DELSYS, USA; sampling frequency: 2000 Hz).

**Figure 1:**
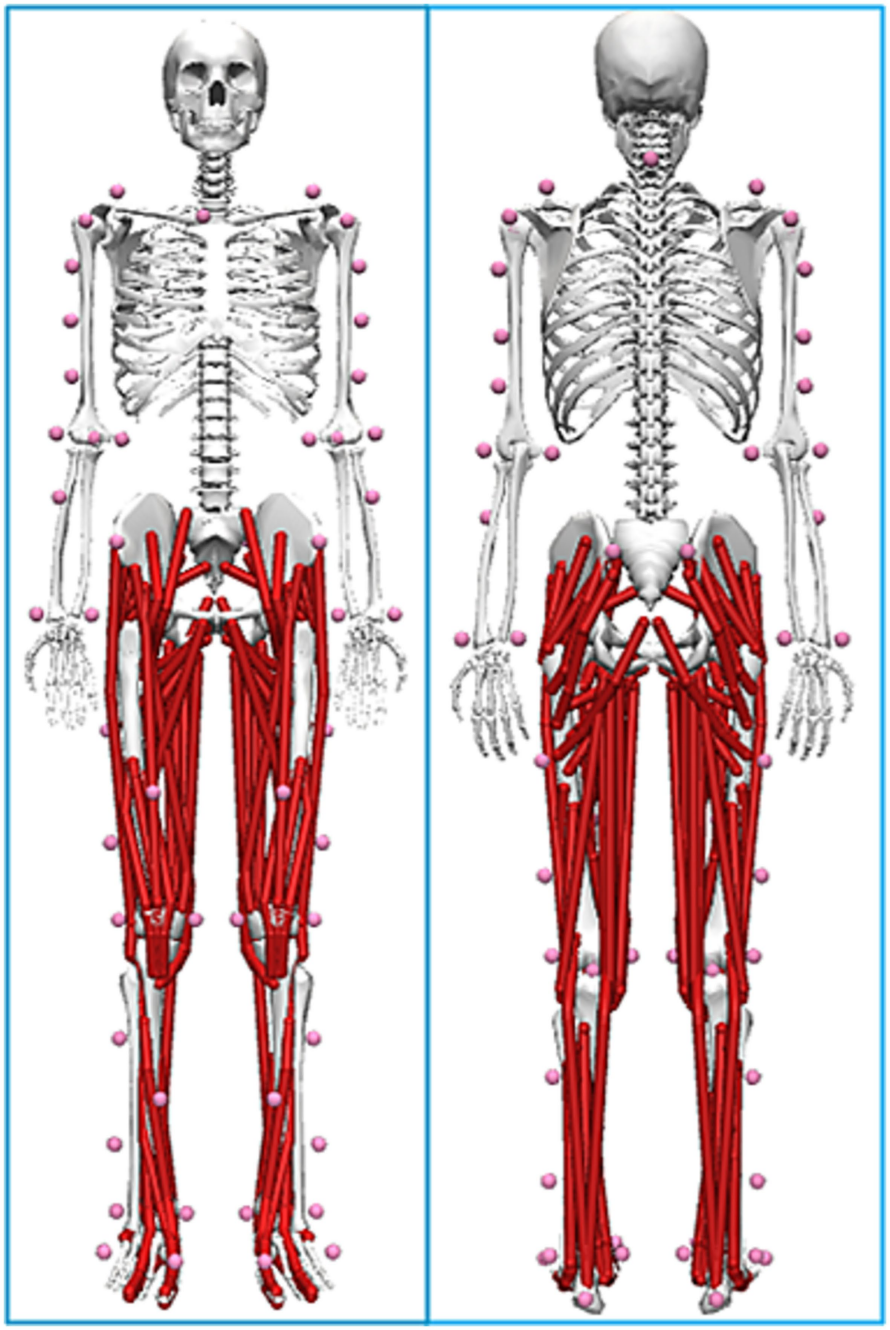
Visualization of the OpenSim Rajagopal marker set used in the study. The marker set protocol was adopted from Rajagopal et al. (2016) to ensure compatibility with the OpenSim musculoskeletal model. The image depicts a participant from the current study.

According to the OpenSim Rajagopal modeling protocol, a total of 56 reflective markers were affixed to the participants’ skin or tight-fitting clothing, with locations as shown in Figure 1 (Rajagopal et al., 2016). Before EMG collection, the skin was degreased with medical alcohol; electrodes and wireless surface EMG sensors were placed on the center of the muscle belly along the direction of the muscle fibers and secured with medical tape. The electrodes were placed according to SENIAM guidelines (Hermens et al., 2000). Sensors were placed on 8 sites in total, covering the bilateral latissimus dorsi (LD), rectus abdominis (RA), erector spinae (ES), and external oblique (EO) muscles, with locations shown in Figure 1.

The testing procedure was as follows: Participants first familiarized themselves with the testing procedure and completed a thorough warm-up. The order of the two asanas (ST and STT) was randomly assigned among participants to eliminate order effects. Within the designated testing area, participants performed the standard left-sided version of each asana according to the “Fitness Yoga Asana Standards” (Social Sports Guidance Center, General Administration of Sport of China, 2018). The core requirements were to maintain axial extension of the spine throughout the twist and to ensure that rotation occurred primarily in the thoracic spine segment. Three valid trials were required for each asana. In each trial, participants were required to hold the final posture statically for 5 s. A 30-s rest was provided between repetitions, and a 2-min rest was provided between the two asanas to ensure adequate recovery.

### 2.3 Maximum voluntary isometric contraction (MVIC) test

To normalize the electromyography (EMG) signals, the MVIC of each target muscle was measured before the formal experiment. Under supervision, participants sequentially performed specific movements including trunk flexion, extension, lateral bending, and rotation in a standing position, contracting with maximum effort against a fixed resistance provided by inelastic straps. Each contraction was held for 5 s and repeated 3 times, with a 2-min rest period between trials. The maximum root mean square (RMS) value from a 1-s stable period across the three trials was used as the MVIC for each muscle.

### 2.4 Modeling, simulation, and data processing

**Figure 2:**
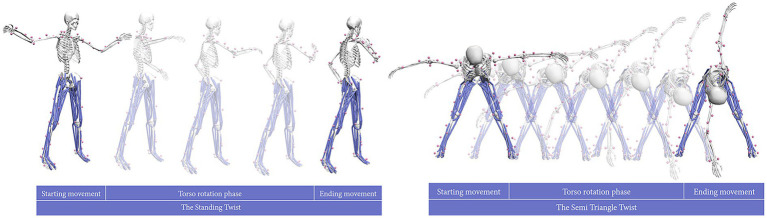
The standing twist (ST) and the semi-triangle twist (STT) OpenSim simulation results. ST, standing twist; STT, semi-triangle twist.

Modeling, simulation, and data processing were all performed in OpenSim 4.3 software. For the analysis, the most stable middle 3 s of data were extracted from the 5-s static holding phase of each asana. The workflow was as follows: C3D files exported from Qualisys were processed by a custom MATLAB script (c3dExport.m) to convert the coordinate system and generate marker trajectory (.trc) files; a fourth-order10 Hz low-pass Butterworth filter was applied to the raw marker trajectories, and the filtered data were then used for the subsequent inverse kinematics (IK) analysis. The simulation utilized the Rajagopal model, which includes 12 segments, 37 degrees of freedom, and 80 muscle actuators. The OpenSim workflow was as follows: (1) Scale the model using a static trial to match the participant’s anthropometric parameters; (2) Perform IK on the scaled model to calculate joint angles; (3) Apply the IK results to the scaled model and perform an inverse dynamics (ID) analysis to obtain joint moment data. The final successful simulation results for this experiment are shown in Figure 2. To evaluate the kinematic accuracy of the musculoskeletal model, we quantified the marker tracking errors from the Inverse Kinematics (IK) analysis. The Root Mean Square (RMS) and maximum errors were calculated across the entire movement cycle for all participants to assess the quality of model scaling and motion reconstruction. The coordinate system convention for trunk kinematics followed the standard definition of the OpenSim model (RajagopalLaiUhlrich2023), where left axial rotation was defined as positive (+) and right axial rotation as negative (−). For kinetics, consistent with the inverse dynamics output of the model, the trunk rotation moments generated during these left-sided tasks were defined and reported as negative values (−).

### 2.5 Surface electromyography (sEMG) data processing

**Figure 3:**
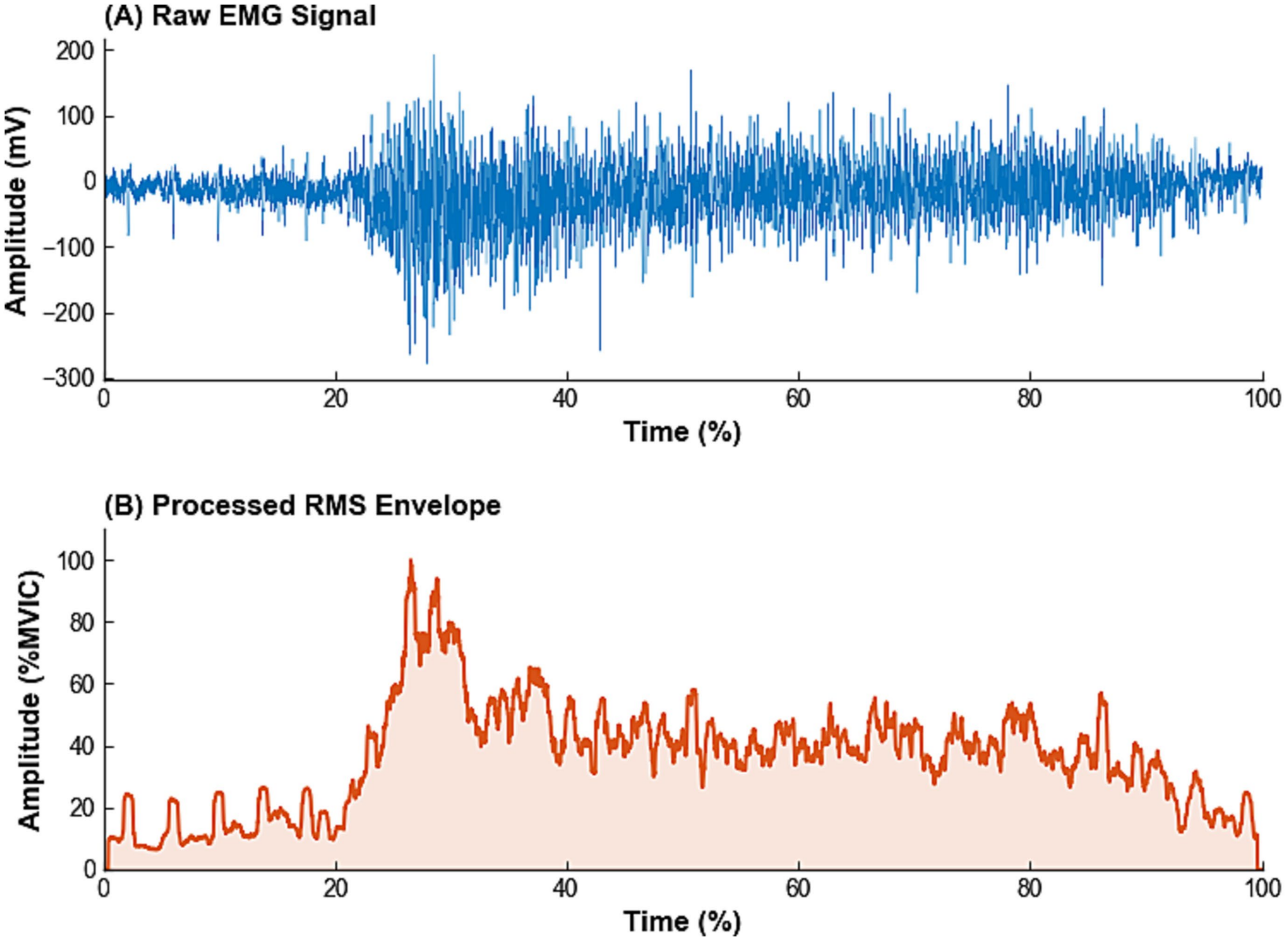
Representative time-domain surface electromyography (sEMG) signals of the left erector spinae (LES) during the semi-triangle twist (STT) pose. **(A)** Raw sEMG signal (mV), showing the physiological burst characteristics. **(B)** Processed RMS envelope (%MVIC). LES, left erector spinae; STT, semi-triangle twist; RMS, root mean square; MVIC, maximum voluntary isometric contraction.

The sEMG signals were processed offline using MATLAB (R2021a, MathWorks, United States) with the following steps: (1) A fourth-order Butterworth band-pass filter (10–500 Hz) was applied to remove noise such as motion artifacts; (2) a 50 Hz notch filter was used to eliminate power-line interference; (3) the signal was full-wave rectified; (4) the root mean square (RMS) value of the rectified signal was calculated and normalized to the MVIC; and (5) the average RMS value within the 3-s analysis window of each asana was taken as the representative value for that trial. The effectiveness of this processing pipeline is visually demonstrated in Figure 3, which compares the raw sEMG signal with the final processed RMS envelope.

### 2.6 Lumbar co-activation ratio

According to functional anatomy (Granata et al., 2005), during leftward rotation, the left erector spinae (LES) and right external oblique (REO) were the agonist muscles, while the right erector spinae (RES) and left external oblique (LEO) were the antagonist muscles. The co-activation ratio was defined as: Co-activation Ratio = RMS_antagonist/RMS_agonist where RMS represents the mean RMS amplitude calculated over the entire movement cycle. That is: Co-activation Ratio = (RMS_RES + RMS_LEO) / (RMS_LES + RMS_REO).

### 2.7 Main outcome measures

Kinematics: Peak trunk rotation angle. Kinetics: Peak trunk rotation moment. Electromyography (EMG): The %MVIC-RMS of the latissimus dorsi, erector spinae, rectus abdominis, and external oblique muscles, and the lumbar co-activation ratio.

### 2.8 Statistical analysis

Data are presented as mean ± standard deviation, and statistical analyses were performed using IBM SPSS 27. A 2 (level: novice, expert) × 2 (asana: ST, STT) mixed design was employed, with “level” as the between-subjects factor and “asana” as the within-subjects factor. To strictly control the Type I error rate inflation due to multiple dependent variables, the 11 analysis variables were divided into three functional families based on their physiological relationships: the Mechanical family (2 variables: peak trunk rotation angle and peak trunk rotation torque), the EMG family (8 variables: activation levels (%MVIC) of eight trunk muscles), and the Co-activation family (1 variable: lumbar co-activation ratio). The Holm-Bonferroni correction method was applied within each family to re-evaluate the statistical significance of the *p*-values. Prior to conducting the ANOVA, the data from the three valid trials for each participant were averaged. The normality of the data was assessed using the Shapiro–Wilk test, and sphericity was verified by Mauchly’s test. When a significant interaction was detected, a simple effects analysis was conducted. Effect sizes were calculated for all ANOVA results to evaluate the magnitude of the effects. The initial significance level was set at *α* = 0.05 and was subject to the aforementioned corrections.

## 3 Results

### 3.1 Kinematic and kinetic characteristics

**Figure 4:**
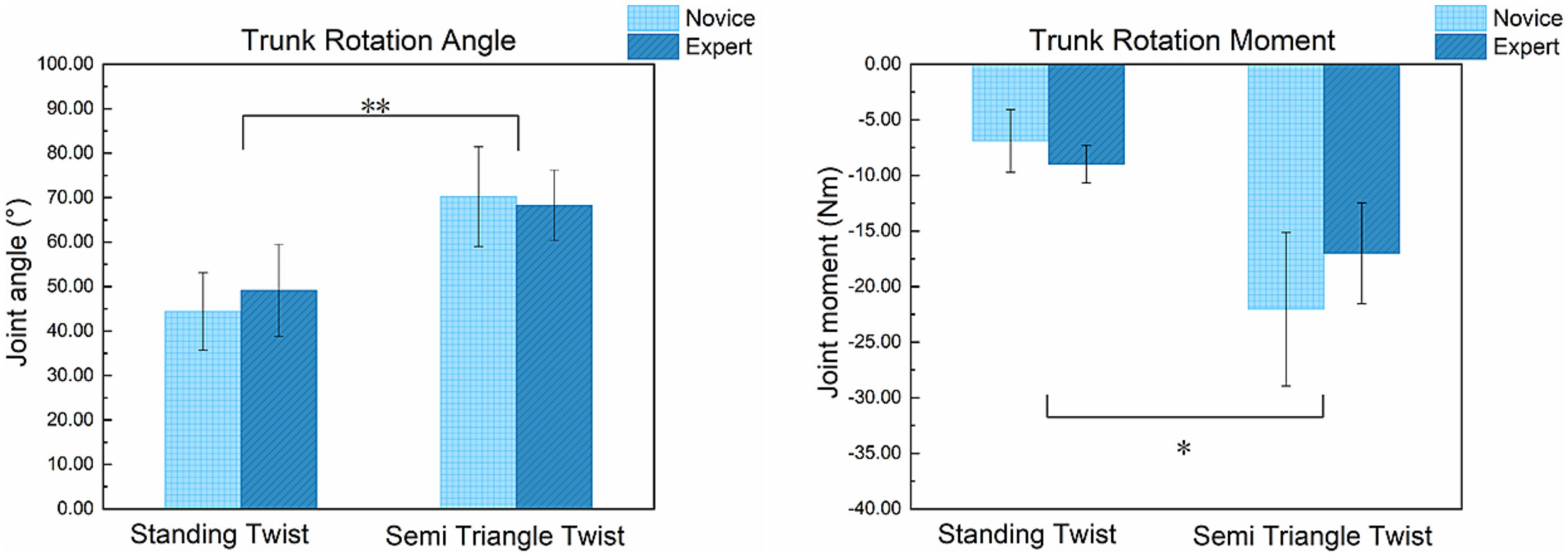
Comparison of peak kinematic and kinetic values between different skill levels and asanas. ** indicates statistical significance (*p* < 0.001) and * indicates statistical significance (*p* < 0.05) after applying Holm–Bonferroni correction.

The descriptive statistics are presented in Table 1 and visually summarized in Figure 4. The ANOVA results (Table 2) show a significant main effect of asana on both kinematic and kinetic variables after applying the Holm-Bonferroni correction. Specifically, compared to the ST asana, the STT asana induced a significantly greater peak trunk rotation angle (*F* = 38.63, *p* < 0.001) and a significantly higher peak trunk rotation moment (*F* = 13.92, *p* = 0.020).

**Table 1:** Descriptive statistics (mean ± standard deviation) for trunk rotation angle and moment by asana and experience level.

Variable	Level	ST	STT
Trunk rotation angle (°)	Novice	44.40 ± 8.71	70.24 ± 11.21
	Expert	49.13 ± 10.35	68.29 ± 7.92
Trunk rotation moment (Nm)	Novice	−6.89 ± 2.82	−22.04 ± 6.91
	Expert	−8.98 ± 1.68	−17.01 ± 4.54

Sign convention: (+) indicates left rotation, (−) indicates right rotation. ST, standing twist; STT, semi-triangle twist.

**Table 2:** Results of the two-way mixed-design ANOVA for trunk rotation angle and moment.

Variable	Effect	*F*	*p*	*η* ^2^
Trunk rotation angle	Level	0.03	0.875	0.001
	Asana	38.63	<0.001*	0.547
	Interaction	0.33	0.584	0.010
Trunk rotation moment	Level	0.23	0.655	0.007
	Asana	13.92	0.020*	0.303
	Interaction	1.28	0.301	0.038

*p < 0.05 indicates significance after Holm–Bonferroni correction for the mechanical variable family. ANOVA, analysis of variance.

However, the main effect of level and its interaction with asana were both non-significant. No statistically significant differences were found between the expert and novice groups for either rotation angle (*p* = 0.875) or rotation moment (*p* = 0.655). The results of the interaction analysis (angle: *p* = 0.584; moment: *p* = 0.932) indicate that the effect of asana on performance was similar across both skill groups.

### 3.2 Muscle activation characteristics

#### 3.2.1 Lumbar co-activation ratio

**Figure 5:**
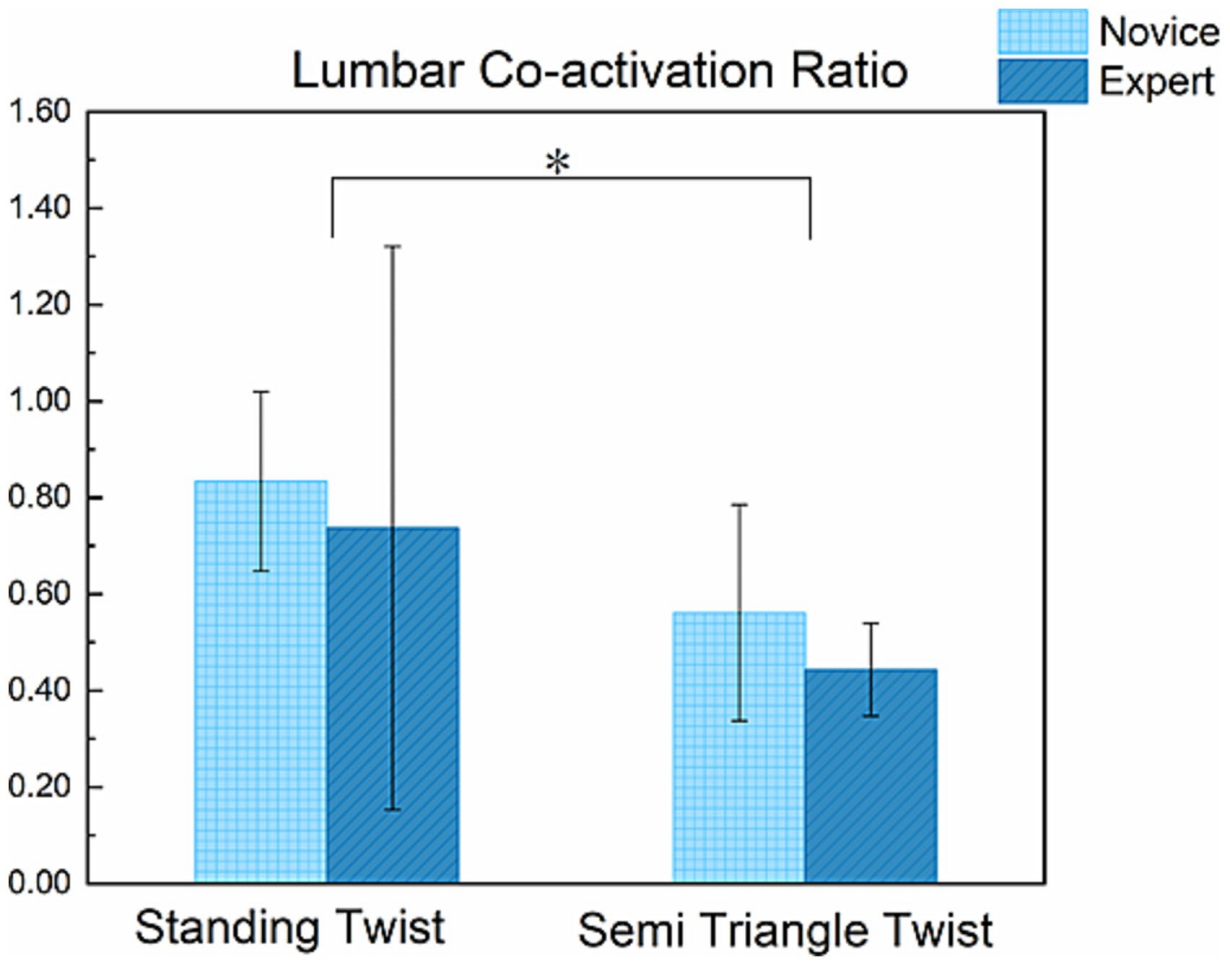
Comparison of the lumbar co-activation ratio between different asanas and skill levels. * indicates a statistically significant main effect of asana (*p* < 0.05).

The descriptive statistics are presented in Table 3 and Figure 5, and the ANOVA results (Table 4) reveal a significant main effect for asana in the lumbar co-activation ratio (*F* = 11.48, *p* = 0.015), which serves as a comprehensive index of neuromuscular control efficiency and joint stability. A key finding was that the co-activation ratio for the ST asana was significantly higher than for the STT asana (Table 3), indicating that muscular coordination was paradoxically more efficient during the more difficult STT, with less inhibitory action from antagonist muscles. The main effect of level (*p* = 0.318) and the interaction effect (*p* = 0.301) were both non-significant.

**Table 3:** Descriptive statistics (mean ± standard deviation) for the lumbar co-activation ratio by asana and experience level.

Variable	Level	ST	STT
Lumbar co-activation ratio	Novice	0.834 ± 0.186	0.561 ± 0.224
	Expert	0.737 ± 0.584	0.443 ± 0.096

**Table 4:** Results of the two-way mixed-design ANOVA for the lumbar co-activation ratio.

Variable	Effect	*F*	*p*	*η* ^2^
Lumbar co-activation ratio	Level	1.18	0.318	0.034
	Asana	11.48	0.015*	0.264
	Interaction	1.28	0.301	0.037

*p < 0.05 indicates significance. ANOVA, analysis of variance.

#### 3.2.2 Activation characteristics of individual trunk muscles

**Figure 6:**
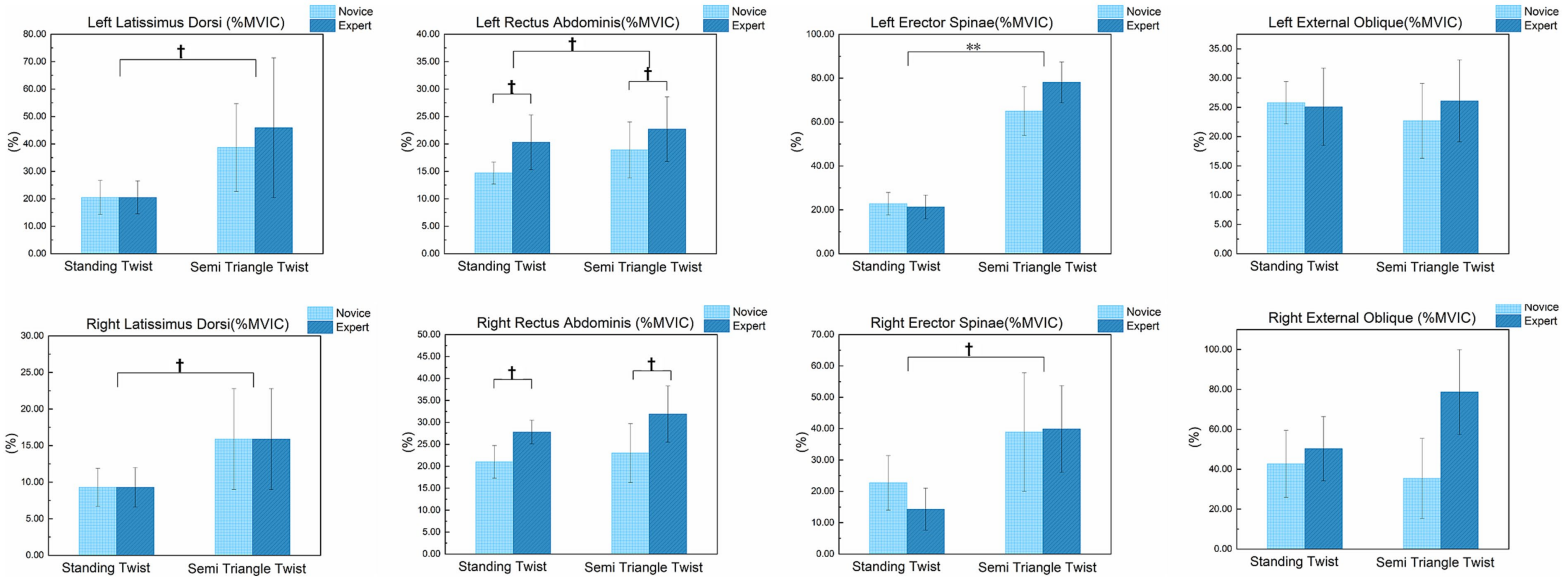
The effect of asana and skill level on the activation levels of key trunk muscle groups. ** indicates statistical significance (*p* < 0.01 after Holm–Bonferroni correction). † indicates a notable physiological trend where the unadjusted *p* < 0.05 but did not meet the corrected threshold. LLD, left latissimus dorsi; RLD, right latissimus dorsi; LRA, left rectus abdominis; RRA, right rectus abdominis; LES, left erector spinae; RES, right erector spinae; LEO, left external oblique; REO, right external oblique; MVIC, maximum voluntary isometric contraction.

The descriptive statistics and ANOVA results for the activation levels of the eight core trunk muscles are presented in Tables 5, 6 and Figure 6, respectively. The ANOVA results for the Main Effect of Asana (Table 6) indicate that, after applying the Holm-Bonferroni correction, muscle activation patterns presented distinct characteristics. The analysis results showed that the STT asana induced a statistically significant increase in the activation level of the left erector spinae (LES) compared to the ST asana (*F* = 208.77, *p* < 0.001). Meanwhile, the left latissimus dorsi (LLD; *F* = 10.21, *p* = 0.024), right latissimus dorsi (RLD; *F* = 10.21, *p* = 0.024), left rectus abdominis (LRA; *F* = 5.98, *p* = 0.049), and right erector spinae (RES; *F* = 6.01, *p* = 0.049) showed notable increasing trends (unadjusted *p* < 0.05), although they did not reach the adjusted statistical significance threshold. Among these, the activation of the left erector spinae (LES) showed a particularly dramatic increase during STT, clearly revealing the substantial biomechanical demand for maintaining spinal stability when twisting from a forward-flexed trunk position.

**Table 5:** Descriptive statistics (mean ± standard deviation) for individual trunk muscle activation by asana and skill level.

Variable	Level	ST	STT
LLD (%MVIC)	Novice	20.5 ± 6.2	38.7 ± 16.0
	Expert	20.5 ± 6.0	45.9 ± 25.5
RLD (%MVIC)	Novice	9.3 ± 2.6	15.9 ± 6.9
	Expert	9.3 ± 2.7	15.9 ± 6.9
LRA (%MVIC)	Novice	14.7 ± 2.0	18.9 ± 5.1
	Expert	20.3 ± 5.0	22.7 ± 5.9
RRA (%MVIC)	Novice	21.0 ± 3.7	23.0 ± 6.7
	Expert	27.8 ± 2.7	31.9 ± 6.4
LES (%MVIC)	Novice	22.8 ± 5.1	65.0 ± 11.1
	Expert	21.3 ± 5.4	78.1 ± 9.3
RES (%MVIC)	Novice	22.7 ± 8.7	38.9 ± 18.9
	Expert	14.3 ± 6.7	39.9 ± 13.8
LEO (%MVIC)	Novice	25.8 ± 3.6	22.7 ± 6.4
	Expert	25.1 ± 6.6	26.1 ± 7.0
REO (%MVIC)	Novice	42.7 ± 16.8	35.4 ± 20.1
	Expert	50.3 ± 16.1	78.7 ± 21.2

LLD, left latissimus dorsi; RLD, right latissimus dorsi; LRA, left rectus abdominis; RRA, right rectus abdominis; LES, left erector spinae; RES, right erector spinae; LEO, left external oblique; REO, right external oblique; MVIC, maximum voluntary isometric contraction.

**Table 6:** Results of the two-way mixed-design ANOVA for individual trunk muscle activation by asana and skill level.

Effect	Variable	*F*	*p*	*η* ^2^	Variable	*F*	*p*	*η* ^2^
Level	LLD	0.02	0.981	<0.001	RLD	0.15	1.000	0.242
Asana		10.21	0.024†	0.242		10.21	0.024†	<0.001
Interaction		0.44	0.533	0.014		0.35	1.000	<0.001
Level	LRA	6.64	0.042†	0.172	RRA	11.23	0.015†	0.260
Asana		5.98	0.049†	0.157		5.70	0.053	0.151
Interaction		0.01	0.912	<0.001		0.01	0.941	<0.001
Level	LES	2.12	0.196	0.062	RES	1.18	0.318	0.036
Asana		208.77	<0.001**	0.867		6.01	0.049†	0.158
Interaction		0.93	0.373	0.028		1.40	0.281	0.042
Level	LEO	0.01	0.912	<0.001	REO	1.69	0.243	0.043
Asana		0.23	0.963	<0.001		1.45	0.274	0.050
Interaction		0.28	0.617	0.009		2.22	0.187	0.065

** indicates statistical significance after Holm–Bonferroni correction. † indicates a significant trend based on the unadjusted *p*-value (*p* < 0.05). LLD, left latissimus dorsi; RLD, right latissimus dorsi; LRA, left rectus abdominis; RRA, right rectus abdominis; LES, left erector spinae; RES, right erector spinae; LEO, left external oblique; REO, right external oblique; MVIC, maximum voluntary isometric contraction, ANOVA, analysis of variance.

The ANOVA results for the Main Effect of Leve (Table 6) show that differences in expertise were primarily manifested in the recruitment of the abdominal core musculature. The expert group showed a distinct trend toward higher activation levels than the novice group in both the left rectus abdominis (LRA; *F* = 6.64, *p* = 0.042) and the right rectus abdominis (RRA; *F* = 11.23, *p* = 0.015). This medium-to-large effect sizes suggests that expert practitioners may be more adept at utilizing the abdominal musculature to drive and stabilize the twisting motion.

Regarding the Interaction Effect, for all muscles analyzed, no significant interaction was found between asana and level (all *p* > 0.05). This indicates that the advantage in muscle activation patterns shown by the expert group (particularly in the abdominal muscles) and the different muscular demands imposed by each asana are two relatively independent and stable phenomena.

## 4 Discussion

The core findings of this study can be summarized in two points: First, the transition from ST to STT significantly alters the mechanical landscape, necessitating a substantial reconfiguration of neuromuscular coordination patterns. Second, the difference between expert and novice practitioners is primarily characterized by internal motor control strategies rather than external kinematic outcomes, reflecting a high degree of motor expertise. These results underscore that motor expertise in yoga is manifested through an optimized neuromotor strategy that effectively navigates the stability-flexibility trade-off inherent in complex spinal rotations.

### 4.1 Biomechanical differences and muscle activation patterns between ST and STT

It was observed that as the trunk rotation angle increased, the corresponding moment also increased, which was especially prominent in the STT. The STT is performed in a position of hip flexion, placing greater demands on the flexibility of the soft tissues surrounding the spine and on muscle strength. As the angle of rotation increases, the moment applied by the muscles to the spinal functional unit (adjacent vertebrae, intervertebral disks, facet joints, ligaments, and muscle groups) increases, which significantly elevates the muscular load on the spine (Granata et al., 2005). Our analysis revealed that the STT asana imposes a substantially higher biomechanical and neuromuscular demand than the ST asana. Specifically, the neuromuscular recruitment of the lumbar erector spinae (LES) was significantly elevated, while other synergistic core muscles—including the latissimus dorsi and rectus abdominis—exhibited discernible upward trends in activation. Previous research indicates that the segmental range of motion in degenerated intervertebral disks is greater than in healthy disks, suggesting that excessive rotation angles may not be beneficial and should be kept within a physiological range to foster a balanced development of spinal stability and flexibility (Haughton et al., 2002). In contrast, ST is characterized by a lower rotational moment and a reduced mechanical load on the intervertebral disks, making it a more appropriate introductory pose for novice practitioners. Furthermore, this study identified an “efficiency paradox”: although STT presents higher task complexity and strength requirements, it paradoxically exhibited a significantly lower lumbar co-activation ratio compared to ST, suggesting a shift toward optimized neural efficiency. When task difficulty reaches a certain threshold, the nervous system may more effectively inhibit antagonist activity to concentrate resources on the concentric contraction of the agonists, thereby exhibiting greater coordination efficiency; in the less demanding ST, however, the body might adopt a strategy of higher co-activation to increase joint stiffness and ensure safety.

### 4.2 Neuromuscular control strategies of practitioners at different skill levels

An important finding of this study is that the differences between the expert and novice groups were primarily manifested in their muscle activation patterns, rather than in their kinematic performance. Although no significant differences were found in the terminal rotation angles or moments, the internal control strategies—or “methods”—employed to achieve these movements differed distinctly between the two groups. The core distinction in motor expertise was primarily reflected in the neuromuscular recruitment of the abdominal musculature. The results showed that the activation levels of both the left and right rectus abdominis (LRA, RRA) in the expert group exhibited a consistent trend of being higher than in the novice group. Although these differences did not reach statistical significance after applying the rigorous Holm-Bonferroni correction, the medium-to-large effect sizes suggest that expert practitioners are more adept at using their core abdominal muscles to drive and stabilize complex twisting movements, which may be a key biomechanical marker for distinguishing practitioner skill levels.

The co-activation ratio allows for the analysis of the activation characteristics of agonist and antagonist muscles surrounding a joint, and their co-activation can enhance joint stability (Baratta et al., 1988). In the present study, although the ANOVA revealed that the main effect for group on the co-activation ratio was not statistically significant (*p* = 0.318), the descriptive data (Table 3) still indicated a trend toward a lower co-activation ratio in the expert group. This may suggest that the expert group employed a more efficient and better-coordinated neuromuscular control pattern, more precisely inhibiting unnecessary antagonist muscle activation while performing the agonist contraction. However, this conclusion requires further validation with a larger sample size. Furthermore, the relationship between spinal biomechanics and muscle activity indicates that while muscle strength is negatively correlated with intervertebral disk loading (Hu et al., 2025), an excessive increase in muscle force can reduce spinal mobility, thereby increasing spinal load (Mayer, 1997; Smith and Kulig, 2016). In the long term, this could adversely affect spinal health.

### 4.3 Implications for yoga practice

The findings of this study establish a critical biomechanical and neuromuscular foundation for the safe and effective execution of yoga twisting poses. The STT requires performing the twist from a position of hip flexion, which demands significant flexibility in the spine and hip joints. It is recommended that novices or individuals with insufficient flexibility use props like yoga blocks to elevate their hips, thereby reducing the degree of hip flexion and allowing the spine to maintain its natural extension throughout the twist. Biomechanical studies have confirmed that pure axial rotation places relatively little pressure on the intervertebral disks (Liebsch and Wilke, 2025), whereas twisting combined with improper flexion or lateral bending generates multi-directional composite stresses (tension, compression, and shear), significantly increasing the risk of disk injury (Hou et al., 2023). This study’s finding of significantly increased rotational moment and erector spinae activation during STT provides data to support the safety principle of avoiding deep twists while the spine is in a flexed state. Furthermore, as twisting and flexion increase compressive loads on the posterior and anterior aspects of the intervertebral disks, respectively (Xu et al., 2022), practitioners should perform complex poses like STT slowly and with deep, controlled breathing to maximally protect the disks.

### 4.4 Limitations

This study has the following limitations: (1) the simulation model utilized a generic scaling of the Rajagopal model. To validate the kinematic accuracy, we quantitatively evaluated the marker tracking errors across all 34 participants. The average Root Mean Square (RMS) marker error was 2.50 ± 1.05 cm, and the average maximum marker error was 8.72 ± 2.43 cm. Although the RMS value is marginally higher than the strict threshold typically recommended for gait analysis (<2.0 cm), it reflects the inherent challenge of tracking markers during high-amplitude yoga postures involving substantial skin deformation. Importantly, our analysis revealed that the peak marker errors were consistently localized to the upper extremities (e.g., upper arm markers) due to soft tissue artifacts during extreme arm extension. However, given the substantial trunk rotation (44°–70°) and extensive upper limb trajectories in the Semi-Triangle Twist, these errors yield a low error-to-signal ratio. This indicates that the model successfully captured the gross kinematic patterns despite localized soft tissue artifacts. (2) The simulation model treated the spine as a single rigid body, which prevented segmental analysis. Future research should employ a multi-segment spinal model to conduct a more in-depth investigation of intervertebral kinematics and kinetics. (3) Subcutaneous fat thickness was not measured, which may have influenced the sEMG amplitude; this is a factor that could be considered in future studies. (4) As all participants were female, these findings may not generalize to male practitioners due to inherent biomechanical differences between genders. (5) Furthermore, model parameters were not dynamically optimized (e.g., via Residual Reduction Algorithm) due to convergence challenges inherent in high-amplitude static postures. Future studies could explore advanced optimization frameworks to further enhance dynamic consistency.

## 5 Conclusion

Integrating biomechanical and electromyographic analyses of two yoga twisting poses, this study draws the following core conclusions: Asana-related Differences: Significant biomechanical differences exist between ST and STT. As an advanced pose, STT places significantly higher demands on flexibility, strength, and stability, which is demonstrated by a larger rotational moment and higher activation levels in several core muscles (especially the erector spinae). Intriguingly, the more demanding STT paradoxically exhibited a lower lumbar co-activation ratio, suggesting a transition toward enhanced neuromuscular coordination efficiency. Skill-specific Differences: The fundamental distinction in motor expertise lies in the internal neuromuscular control strategies rather than observable kinematic performance. Despite comparable rotation angles and moments, experts exhibited a distinct trend toward heightened rectus abdominis recruitment, reflecting a superior capacity to utilize core musculature to stabilize and drive the trunk—a key neuromuscular marker of proficiency. In summary, yoga practitioners should select twisting asanas tailored to their individual functional capacities, prioritizing the stability-flexibility trade-off to facilitate the integrated advancement of muscular strength, neuromuscular coordination, and soft tissue mobility.

## Data Availability

The raw data supporting the conclusions of this article will be made available by the authors, without undue reservation.
